# Does early surgery result in improved long-term survival compared to watchful waiting in patients with asymptomatic severe aortic regurgitation with preserved ejection fraction?

**DOI:** 10.1093/icvts/ivac080

**Published:** 2022-03-30

**Authors:** Samuel Heuts, Michal J Kawczynski, J G Maessen, Elham Bidar

**Affiliations:** 1 Department of Cardiothoracic Surgery, Maastricht University Medical Centre, Maastricht, the Netherlands; 2 Cardiovascular Research Institute Maastricht (CARIM), Maastricht University, Maastricht, the Netherlands

**Keywords:** Aortic valve regurgitation, Aortic valve insufficiency, Asymptomatic, Early surgery

## Abstract

A best evidence topic in cardiac surgery was written according to a structured protocol. The question addressed was: *In patients with asymptomatic severe aortic regurgitation with preserved ejection fraction, is early surgery superior to watchful waiting in terms of long-term survival?* Altogether, 648 papers were found using the reported search, 3 of which represented the best evidence to answer the clinical question (all level III evidence). The authors, journal, date and country of publication, patient group studied, study type, relevant outcomes and results of these papers are tabulated. The 3 included studies comprised 469 patients. All 3 studies attempted to correct for potential baseline differences by different matching methods. As a result, a predominantly beneficial effect of early surgery on long-term survival in patients with severe asymptomatic AR and preserved LV function was observed, whereas none of the studies demonstrated a disadvantageous effect. Still, because many of the initially conservatively treated patients eventually proceed to surgery, longer term follow-up is warranted. Of note, older patients especially seem to adapt more poorly to chronic volume overload due to aortic regurgitation, making them potential candidates for a more aggressive approach. However, when a justified watchful waiting strategy is applied, close, extensive monitoring seems to be imperative, because the development of class I and II triggers seems to lead to improved survival.

## INTRODUCTION

A best evidence topic (BET) was constructed according to a structured protocol. This procedure is fully described in Interactive CardioVascular and Thoracic Surgery [1]. The current BET was registered on http://www.bestbets.org (registration 15 January 2022).

## THREE-PART QUESTION

In [patients with asymptomatic severe aortic regurgitation with preserved ejection fraction], is [early surgery superior to watchful waiting] in terms of [long-term survival]?

## CLINICAL SCENARIO

A 53-year-old male patient, with known mild aortic regurgitation (AR), is regularly monitored for follow-up of progression of aortic disease. At the present echocardiographic evaluation, AR progressed and is classified as severe, whereas the left ventricular ejection fraction (LVEF) is 58%, and the left ventricular end systolic diameter (LVESD) is 44 mm [indexed (LVESD) 20 mm/m^2^]. Clinically, the patient is truly asymptomatic and is able to perform intensive exercise. The patient is referred to the multidisciplinary heart valve team for clinical decision making. Although valvular heart disease guidelines recommend follow-up and watchful waiting in these specific patients, some studies have reported conflicting results in a similar patient population when comparing early surgery to a watchful waiting strategy. Therefore, in the current BET, a comprehensive literature review was performed to evaluate long-term survival following early surgery versus watchful waiting in these patients.

## SEARCH STRATEGY

Eligible articles were identified through searching electronic scientific databases including PubMed and the EMBASE library. The following search strategy was used: [((aortic valve insufficiency OR aortic valve regurgitation OR aortic valve incompetence) AND asymptomatic disease AND (cardiac surgical procedures OR cardiac surg* OR sternotomy OR aortic valve replacement OR early surg* OR early operation*))]. References of the articles included were screened for additional eligible papers. Of note, studies describing aortic valve replacement (AVR) and aortic valve repair were all eligible for inclusion.

## SEARCH OUTCOME

In total, 648 papers were found using the aforementioned search. From these, 3 papers were identified that provided the best evidence to answer the question. The included articles are presented in Table 1.

**Table 1:** Best evidence papers

Author, date, journal and country, study type (level of evidence)	Patient groups	Outcomes	Key results	Comments
Turk *et al.*, (2010) Ann Thorac Surg, United States, retrospective cohort (level of evidence III) [2]	79 patients (subgroup of LVEF >50% and LVEDD <70 mm, mean FU 4.8 years) Group with early AVR (n = 21, LVEF 67%, LVEDD 58 mm, LVESD 36 mm, 100% replacement, mean age 52 years) Group with no AVR (n = 58, mean age 63 years, LVEF 65%, LVEDD 52 mm, LVESD 33 mm)	1-, 5- and 10-year survival, unmatched (%)	Early AVR: 100%, 94%, 94% No AVR: 86%, 71%, 46%	Subgroup analysis of patients undergoing early AVR versus no AVR. All surgical patients underwent valve replacement exclusively. Older patients included in the no-AVR group. More severe LV dilatation in the early AVR group. Early AVR patients had more intensive medical therapy.
		Hazard ratio for long-term survival, matched	Early AVR: HR 0.11 (P = 0.04)	
		Predictors of late survival (RR, 95% CI)	Early AVR (RR 0.068, 95% CI 0.007-0.673) CKD (RR 3.3, 95% CI 1.2-8.8) COPD (RR 4.1, 95% CI 1.4-12.3) DM (RR 14.1, 95% CI 3.1-64.6)	
De Meester *et al.* (2015), J Thorac Cardiovasc Surg, Belgium, retrospective cohort (level of evidence III) [3]	160 patients (median FU 7.2 years) Early surgery (n = 91, mean age 49 years, BAV n = 51, LVEF 59%, LVEDD 64 mm, LVESD 43 mm, AV-sparing n = 76, Ross procedure n = 7, AVR n = 8) Conservative (n = 69 patients, mean age 50 years, BAV n = 30, LVEF 58%, LVEDD 61 mm, LVESD 40 mm)	5- and 10-year survival, unmatched (%)	Early surgery: 93%, 91% Conservative: 97%, 89%	Majority of patients underwent valve-sparing surgery. Improved survival in patients undergoing regular echocardiographic assessment in the watchful waiting group.
		5- and 10-year survival, matched (%)	Early surgery: 95%, 95% Conservative: 95%, 95%	
		5- and 10-year survival, IPW- adjusted (%)	Early surgery: 93%, 92% Conservative: 99%, 92%	
		Development of class I/II triggers with subsequent surgery during follow-up (%)	Conservative: 42%	
		Reinterventions (%)	Early surgery: 9.9% Conservative: 6.9%	
Wang *et al.* (2017), Eur J Cardiothorac Surg, China, retrospective cohort (level of evidence III) [4]	230 patients (median FU 6.1 years) Early surgery (n = 154, mean age 54 years, BAV n = 14, mean root diameter 45 mm, LVEF 58%, LVEDD 77 mm, LVESD 44 mm 100% AV replacement) Conservative (n = 76, mean age 56 years, BAV n = 3, mean root diameter, 45 mm, LVEF 59%, LVEDD 74 mm, LVESD 43 mm)	3-, 5- and 10-year survival, unmatched (%)	Early surgery: 97%, 93%, 87% Conservative: 92%, 86%, 79%	Included patients had severe LV dilatation (77 mm and 74 mm, respectively). All surgical patients underwent valve replacement exclusively.
		3-, 5- and 10-year survival, matched (%)	Early surgery: 98%, 95%, 90% Conservative: 94%, 87%, 79%	
		Development of class I/II triggers with subsequent surgery (%)	Conservative: 37%	
		Echocardiographic results (6-month LVEDD reduction [mm], LVEF improvement [%])	Early surgery: LVEDD: -15 mm LVEF: (-) Conservative (with late operation): LVEDD: -12 mm; LVEF: -2%	

AV: aortic valve; AVR: aortic valve replacement; BAV: bicuspid aortic valve; CI: confidence interval; CKD: chronic kidney disease; COPD: chronic obstructive pulmonary disease; ; DM: diabetes mellitus; FU: follow-up; HR: hazard ratio; IPW: inverse probability weighting; LV: left ventricle/ventricular; LVEDD: left ventricular end diastolic diameters; LVEF: left ventricular ejection fraction; LVESD: left ventricular end systolic diameter; RR: relative risk.

## RESULTS

All 3 studies included in the current BET comprised retrospective cohorts (level III evidence). In total, the BET incorporates 469 patients with asymptomatic severe AR and preserved LVEF undergoing either early surgery (*n* = 266) or an initially conservative management (*n* = 203). Baseline characteristics, procedural aspects and outcomes are presented in detail in Table 1.

The first study, by Turk *et al.*, was conducted in the United States and describes the period 1993 to 2007 [2]. Although the initially analysed cohort comprised more patients (*n* = 123), a subgroup analysis of patients with *normal* systolic LV function and *non-excessively* dilated LVs was performed (*n* = 79 in total, early AVR: *n* = 21, no AVR: *n* = 58, mean age 52 and 63 years, respectively). Left ventricular function and diameters for the early AVR and no-AVR group were as follows: LVEF 67% and 66%, LVEDD 58 mm, 52 mm and LVESD 36 mm, 33 mm, respectively. Because only the subgrouped data apply to the current BET, these data specifically were included in our interpretation of the best available evidence. Compared to the other included studies, the study by Turk *et al.* describes a markedly older patient population (mean age 60 years). Of note, all surgical patients underwent AVR. An important beneficial effect of an early AVR strategy was observed (1-, 5- and 10-year survival 100%, 94% and 94% versus 86%, 71% and 46% respectively, *P* = 0.004). However, it should be noted that patients in the no-AVR group were older, whereas patients in the early AVR group tended to have more diseased and dilated LVs and received more intensive medical treatment. Still, after adjustment for baseline between-group differences and adjustment for univariate predictors of mortality, early AVR remained an independent predictor of survival (relative risk 0.03, *P* = 0.007). Additionally, the beneficial effect was confirmed in propensity score matched groups (early AVR hazard ratio 0.11, *P* = 0.04). Although the study’s sample size, especially in the early AVR group, was relatively small, a markedly and surprisingly significant effect was found. As such, it should be kept in mind that observed results might be attributed to other factors that were not described and corrected for in this study. Moreover, patients in the conservative group (no AVR) did not seem to proceed to AVR in a later stage, implying this population comprised patients at an increased or prohibitive surgical risk or with more important comorbidities. Although the authors performed a matching analysis to correct for potential confounders, propensity matching only allows for the matching of *known* confounders, leaving the potential of selection bias by *unknown* confounders. Furthermore, in all studies describing *asymptomatic* patients, it remains questionable whether subjects are truly asymptomatic or do develop mild symptoms upon extensive exertion.

The second study, by de Meester *et al.*, was performed in Belgium between 1995 and 2012 [3]. In this retrospective analysis, a comparison between early surgery and an initially conservative treatment in patients with asymptomatic AR and normal systolic LV function and dimensions was performed. The early surgery group consisted of 91 patients [mean age 49 years, presence of bicuspid aortic valve (BAV) *n* = 51 (56%)], the conservative group, of 69 patients [mean age 50 years, BAV *n* = 30 (43%)]. Echocardiographic characteristics, in terms of LVEF, LVEDD and LVESD, were 59% and 58%, 63 and 61 mm and 43 and 40 mm for the early surgery and conservative treatment group, respectively. In the early invasive group, predominantly aortic valve repair (91%) was performed and, to a lesser degree, AVR (9%) [3]. Long-term survival analysis, with a median follow-up of 7.2 years, failed to demonstrate a beneficial effect of an early surgical strategy (5- and 10-year survival 93% and 91% versus 97% and 89% respectively, *P* = 0.87). Multivariate Cox proportional hazards analysis identified age and male gender as independent predictors of survival, whereas early surgical intervention was not associated with survival (*P* = 0.45). Propensity score matching and inverse probability weighting were performed to correct for potential baseline differences between both groups, but the observed results in the overall analysis persisted (5- and 10-year survival was 95%, 95% versus 95%, 95%, *P* = 0.93 and 93%, 92% versus 97%, 92%, *P* = 0.55, respectively). In a more in-depth analysis of the watchful waiting group, patients were stratified into a closely monitored cohort and a more loosely followed-up group. Patients with less follow-up had significantly worse survival than patients monitored closely (95% vs 79%, respectively, *P* = 0.045). Interestingly, 42% of these initially conservatively treated patients eventually required surgery because of the development of class I or II triggers during the follow-up period. De Meester *et al.* was the only group reporting reintervention rate as an outcome, which appeared to be 10% in the early surgery group and 6% in the initially conservatively treated group. Subsequently, the authors concluded that an early surgical strategy is not different from an initially conservative strategy, provided that the latter patient group is monitored extensively and regularly and referred for surgery immediately upon symptom development.

Wang *et al.* studied a patient cohort in China from 2003 to 2014 [4]. The authors compared the results of early surgery [*n* = 154, mean age 54 years, BAV *n* = 14 (9%), mean root diameter 45 mm] to initially conservative treatment [*n* = 76, mean age 56 years, BAV *n* = 3 (4%), mean root diameter 45 mm] in patients with asymptomatic AR, normal systolic LV function *and* left ventricular dilatation (LVEDD >70 mm) [4]. In the surgical cohort, AVR was performed exclusively (mechanical valve prosthesis *n* = 139, biological prosthesis *n* = 15). In the early surgery group, LVEF was 58%, LVEDD 77 mm and LVESD 44 mm, whereas LVEF was 59%, LVEDD 74 mm and LVESD 43 mm in the initially conservative group. Wang *et al.*, in the overall cohort, found that there was a tendency, but not a statistically significant one, towards improved survival using an early surgical strategy regarding *all-cause mortality* (3-, 5- and 10-year survival 97%, 93% and 87% vs 92%, 86% and 79%, respectively, *P* = 0.067). However, when looking specifically at *cardiovascular mortality*, an early invasive strategy was associated with statistically significantly improved survival (*P* = 0.037). In addition, in the initially conservatively treated group, 37% of patients would eventually undergo surgery due to development of class I or II triggers during the follow-up period, at a mean interval of 4.2 years after enrolment. Wang *et al.* also performed a propensity score analysis to correct for baseline differences, in a 2:1 ratio. In the matched analysis, early surgery was associated with improved long-term survival for both all-cause mortality (3-, 5- and 10-year survival 98%, 95% and 90% vs 94%, 87% and 79%, respectively; *P* = 0.018) and cardiovascular mortality (3-, 5- and 10-year survival 98% vs 96%, 94% and 93% and 88% and 80%, *P* = 0.008). Of note, this patient cohort differs from those in the prior 2 studies, because the mean LVEDD was markedly increased (mean LVEDD >75 mm), implying these patients were in a more advanced disease state. As such, the observed beneficial effect of an “early” invasive strategy must be interpreted with caution, because intervention at this stage might not be comparable to, and as “ early”, as the interventions in the other 2 included studies.

The most notable difference in baseline characteristics between the 3 included studies was age, because patients in the study by de Meester *etal.* were markedly younger than patients in the other studies. Because both Turk *et al.* and Wang *et al.* found a beneficial effect of an early invasive strategy, unlike de Meester *et al.*, the current findings might imply a more pronounced treatment effect in older patients with a reduced ability to cope with volume overload associated with AR.

## CLINICAL BOTTOM LINE

The goal of the current BET was to evaluate the potential beneficial effect of an early invasive strategy compared to an initially conservative strategy in asymptomatic patients with severe AR with preserved left ventricular function. We found a predominantly beneficial effect of early surgery on long-term survival in this specific patient population, and none of the studies demonstrated a disadvantageous effect. Moreover, many patients with an initially conservative strategy eventually proceed to AVR during the follow-up period, in a potentially more advanced disease state, which can affect long-term outcome as well. Therefore, even more longer term follow-up than that provided by the included studies is warranted, to more adequately answer this important matter of debate. Furthermore, the presence of BAV and aortic root and ascending aortic diameter are important factors to take into account, the influence of which was not analysed separately in the present studies. Of note, older patients especially seem to adapt more poorly to chronic volume overload due to AR, making them potential candidates for a more aggressive approach. However, when a justified watchful waiting strategy is applied, close, extensive monitoring seems to be imperative, because the mere monitoring for development of class I and II triggers seems to lead to improved survival.
